# Oxygen-enhanced assembloid-based vascularized intestinal-on-a-chip for radioprotective drug evaluation

**DOI:** 10.3389/ftox.2026.1863392

**Published:** 2026-07-16

**Authors:** Yuting Guo, Meiling Fu, Yuan Pang

**Affiliations:** 1 Biomanufacturing Center, Department of Mechanical Engineering, Tsinghua University, Beijing, China; 2 Key Laboratory for Advanced Materials Processing Technology, Ministry of Education, Beijing, China; 3 Beijing Key Laboratory of Intelligent Organ Biofabrication and Regenerative Repair, Tsinghua University, Beijing, China; 4 The State Key Laboratory of Membrane Biology, Tsinghua-Peking Center for Life Sciences, School of Life Sciences, Tsinghua University, Beijing, China

**Keywords:** organ-on-chip, intestine model, stromal microenvironment, vascularized, radiation injury, drug screening

## Abstract

**Background:**

The intestine plays essential roles in digestion, immunity, and metabolism, but is highly sensitive to ionizing radiation during cancer treatment or environmental exposure. Although three-dimensional intestinal models more accurately replicate tissue architecture than conventional monolayers, they are frequently limited by insufficient oxygen delivery, leading to hypoxia-associated functional impairment. Incorporating physiologically relevant oxygenation strategies remains a key challenge in advanced *in vitro* systems. This study aimed to establish a physiologically relevant *in vitro* intestinal model by improving oxygen mass transfer within three-dimensional tissue constructs, thereby enhancing structural organization and functional maturation, and subsequently applying the system to investigate radiation-related mechanisms and evaluate potential protective agents.

**Methods:**

Based on a microengineered 3D cell-assembly platform, intestinal epithelial cells, endothelial cells, and fibroblasts were spatially organized to form a structured intestinal tissue model. The composition of the cells was optimised to improve tissue uniformity and the function of the epithelial barrier. To alleviate hypoxia in the three-dimensional construct, an oxygen-permeable microwell system was employed to enhance oxygen diffusion and promote epithelial differentiation, as evidenced by the upregulation of *Isx*, *Cyp3a5*, and *Tff3*. A vascularized chip that recapitulated the *in vivo* intestinal–stromal–vascular interface was created by incorporating microvascular networks self-assembled from endothelial cells within a hydrogel matrix.

**Results:**

Following radiation exposure, the model exhibited characteristic symptoms of intestinal injury, such as decreased cell viability, impaired E-cadherin signaling, and compromised epithelial barrier integrity. Treatment with dimethyloxalylglycine (DMOG) mitigated these effects, confirming it’s utility for assessing radioprotective drugs.

**Conclusion:**

This vascularized intestinal-on-a-chip model closely mimics the structure and function of the human intestinal microenvironment. It serves as a robust *in vitro* platform for studying radiation-induced intestinal injury and for screening candidate protective agents, offering valuable applications in radiation protection and regenerative medicine research.

## 1 Introduction

The intestine is a multifunctional organ fundamental to digestion, immunity, and metabolism. It governs food breakdown, nutrient uptake, waste excretion, hormonal secretion, and microbial immune defense (Murphy and Bloom, 2006; Peterson and Artis, 2014; Zorn and Wells, 2009). However, intestinal tissue is one of the most radiosensitive organs in the human body. During cancer radiotherapy or accidental exposure to ionizing radiation, the intestinal mucosa can suffer severe structural and functional impairment, including epithelial barrier disruption, inflammation, and loss of absorptive capability. With the growing incidence of gynecologic and colorectal tumors treated by pelvic or abdominal radiotherapy and the increasingly extended patient survival time, radiation-induced intestinal injury has become a major clinical and toxicological concern (Kumagai et al., 2018). Nearly half of pelvic radiotherapy patients report persistent gastrointestinal symptoms collectively referred to as radiation-induced enteropathy, which severely compromises quality of life and is a contributing factor to short bowel syndrome and intestinal failure (Bruneton et al., 1982; Li, 2020). These radiation-associated complications impose a substantial socioeconomic burden, highlighting the urgent need for constructing a structurally biomimetic, functionally robust *in vitro* intestinal tissue model suitable for drug evaluation to screen potential drugs for radiation-induced injury.

Traditional two-dimensional (2D) monolayer cell culture models are structurally simple, highly reproducible, and remain the most commonly used *in vitro* platforms for radiation-related studies (Gao et al., 2024; Park and Yu, 2023). However, the planar arrangement of cells in two-dimensional cultures restricts cell–cell interactions and limits the expression of physiological functions. In contrast, three-dimensional (3D) cellular aggregates better recapitulate the biological complexity of native tissues. Tissue models constructed using these multicellular spheroids as basic units can more faithfully reproduce *in vivo* cell–cell and cell–matrix interactions, signal transduction, and extracellular matrix function (Kharkar et al., 2013). Organoid technology also offers distinct advantages in structural complexity and functional reconstruction, providing an *in vitro* model that more closely mimics the intestinal microenvironment under radiation exposure (Bhanja et al., 2018; Montenegro-Miranda et al., 2020). Nevertheless, intestinal organoids typically display a random spatial organization (Lancaster and Knoblich, 2014), with limited control over cell-type composition. During culture, they often develop hypoxic cores and experience restricted nutrient supply (Simian and Bissell, 2017; Yin et al., 2016), compromising tissue maturation and functional stability. Previous studies have shown that radiation-induced epithelial and DNA damage are primarily mediated by endothelial apoptosis and the generation of reactive oxygen species (Paris et al., 2001; Wang et al., 2007). Most current *in vitro* intestinal models lack functional vascular networks, making it difficult to reproduce physiologically relevant oxygen gradients, nutrient exchange, and bidirectional regulation between the vasculature and epithelium (Folkman and Camphausen, 2001). This limitation also prevents meaningful investigation of radiation-induced injury and other pathological processes involving microvascular regulation. Therefore, achieving controlled cellular assembly, improving oxygen transport and tissue oxygenation, and incorporating vascularized structures to enhance physiological relevance have become critical challenges in current *in vitro* intestinal tissue engineering.

During tissue construction, cell composition, proportions, and the microenvironment for mass transport play critical roles in determining tissue functionality. Within intestinal tissue, mesenchymal cells not only provide structural support for the intestinal epithelium but also secrete extracellular matrix proteins such as fibronectin, as well as various growth factors including bone morphogenetic proteins, which collectively regulate epithelial barrier integrity and homeostasis (Lindemans et al., 2015; Vilardi et al., 2024). Vascular endothelial cells are likewise essential, serving as the primary mediators of oxygen and nutrient transport and playing central roles in tissue repair following injury (Paris, Fuks, Kang, Capodieci, Juan, Ehleiter, Haimovitz-Friedman, Cordon-Cardo and Kolesnick, 2001; Vilardi, Przyborski, Mobbs, Rufini and Tufarelli, 2024; Wang, Boerma, Fu and Hauer-Jensen, 2007). Therefore, establishing a 3D cellular assembly system that incorporates multiple stromal cell types is vital for constructing physiologically functional *in vitro* intestinal models. Currently, the use of honeycomb-like micro-porous arrays to generate 3D cellular clusters has been widely adopted (Lee et al., 2016). However, when high-density cellular aggregates are formed using this technique, tight intercellular connections can lead to excessive oxygen consumption within the clusters. This often results in severe local hypoxia in the culture environment, which ultimately impairs tissue functionality (Tan et al., 2024). Hence, the development of *in vitro* 3D intestinal models featuring a well-defined stromal microenvironment and improved oxygen mass-transfer capability represents a critical step toward enhancing the functional fidelity of engineered intestinal tissues.

**FIGURE 1:**
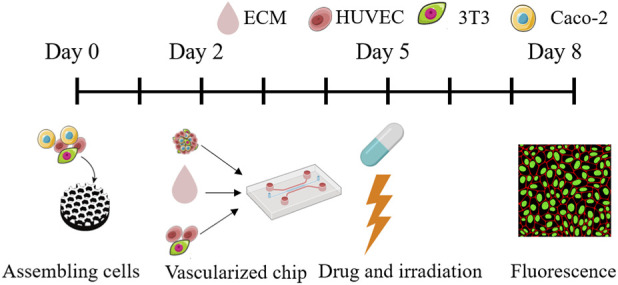
Overview of the experimental platform for intestinal *in vitro* modeling and radiotoxicity evaluation. Workflow for radiation-induced intestinal injury modeling and drug evaluation. Vascularized assembloids were exposed to irradiation within the microfluidic chip, followed by assessment of injury biomarkers and screening of potential radioprotective compounds.

Building upon these considerations, the present study systematically constructed and optimized *in vitro* intestinal tissue models from three key perspectives—stromal microenvironment formation, oxygen-transfer optimization, and vascularization. First, using a honeycomb-array platform, we achieved controlled assembly of mesenchymal cells, vascular endothelial cells, and intestinal epithelial cells, and adjusted the proportion of stromal components to evaluate their effects on tissue morphogenesis and functional development. Subsequently, an improved honeycomb-array system with enhanced oxygen transport capability was employed to provide a more favorable mass-transfer environment for the 3D cellular constructs, enabling investigation of oxygen’s influence on the functional performance of the engineered intestinal model. On this basis, we further integrated vascular-on-chip technology to develop a vascularized intestinal tissue model. Vascular network not only improved oxygen supply but also enhanced the physiological relevance of the model. Finally, the vascularized intestinal construct was applied to radiation-injury drug testing, demonstrating its feasibility and potential utility for simulating radiation-induced damage and evaluating pharmacological interventions (Figure 1).

## 2 Materials and methods

### 2.1 Cell culture

Caco-2 cells (HTB-37, ATCC) were cultured in high-glucose DMEM (Gibco, 11960044) supplemented with 20% (v/v) FBS (Gibco, 10100147) and 1% (v/v) penicillin/streptomycin (Gibco, 15140). HUVECs (DFSC-EC-01, Zhongqiao Xinzhou) were maintained in specialized endothelial-cell medium (ZQ-1304, Zhongqiao Xinzhou), and 3T3 fibroblasts (CRL-1658, ATCC) were cultured in high-glucose DMEM with standard supplements. Cells were subcultured at approximately 80% confluency using 0.05% trypsin-EDTA solution (0.05% trypsin + 0.03% EDTA) at a ratio of 1:3.

### 2.2 Fabrication of spheroid culture systems and oxygen-permeable spheroid culture systems

Honeycomb-structured PDMS membranes (Wistron, Hangzhou, SC600) were fabricated using a standard soft-lithography process. Briefly, a hexagonal photomask pattern with an inscribed circle diameter of 126 μm was designed and transferred onto a silicon wafer by photolithography to create an array of hexagonal prism molds. PDMS prepolymer (Sylgard 184, Dow Corning) mixed with curing agent at a 10:1 ratio was cast onto the mold and thermally cured to form membranes containing honeycomb-shaped micropores (diameter ≈ 126 μm). The cured PDMS sheets were punched into circular discs (15.5 mm in diameter) and fitted into the bottom wells of standard 24-well cell-culture plates for subsequent experiments.

An oxygen-permeable device consisting of honeycomb microwell arrays was fabricated for spheroid cultures, as reported previously (Shinohara et al., 2014). The cured PDMS sheets were punched into circular discs (15.5 mm in diameter) and fitted into the bottom wells of an in-house-designed oxygen-permeable culture plate, which employed a PDMS layer at its bottom to allow direct oxygen diffusion during spheroid culture.

### 2.3 Oxygen concentration simulation

Oxygen diffusion and consumption within the culture system were simulated using COMSOL Multiphysics (v6.0). Oxygen transport was modeled using a transient diffusion equation assuming dilute species transport. Oxygen consumption within organoids was described using a Michaelis–Menten kinetic model.

The oxygen diffusion coefficient was set to 3 × 10^−9^ m^2^/s in the culture medium and 4 × 10^−9^ m^2^/s in PDMS, according to literature values. The maximal oxygen consumption rate was defined as 0.0021 mol/(m^3^·s), with a Michaelis constant of 0.1 mol/m^3^. The initial oxygen concentration was set to 0.194 mol/m^3^, corresponding to equilibrium with atmospheric oxygen. The culture medium height was 6 mm and the PDMS thickness was 1.5 mm (Grauso et al., 2019, Magliaro et al., 2019).

### 2.4 Cluster morphology analysis

The morphology of the Caco-2 cell cluster was observed using a phase-contrast microscope (Eclipse TS100, Nikon, Tokyo, Japan). The average diameter and circularity of Caco-2 cultures were measured and analyzed using ImageJ software. The circularity of Caco-2 culture was calculated using the following formula:

$$Circularity=\frac{4\pi A}{P^{2}}$$

where A is the cross-sectional area of the Caco-2 spheroid, and p is the cross-sectional perimeter of the Caco-2 spheroid.

### 2.5 Microfluidic chip fabrication

Chip patterns were milled using a CNC micro-engraving machine. PDMS membranes were cut into 45 mm × 15 mm rectangular strips for assembly. Both polydimethylsiloxane (PDMS; Sylgard 184, Dow Corning) and polymethyl methacrylate (PMMA; purchased from a local supplier in Suzhou, China), components were soaked in ethanol for 3 min, rinsed three times with distilled water, and dried at 50 °C for 20 min. Plasma treatment was then applied for 120 s on both materials, followed by silanization of PMMA in 5% (APTES) coupling-agent solution for 10 min at 80 °C.

After secondary plasma activation (30 s), PDMS and PMMA layers were immediately bonded. To reinforce sealing, 5 g PDMS prepolymer (10:1 ratio) was injected with a syringe to fill the interface gaps, followed by curing at 70 °C for 1 h and storage at room temperature.

### 2.6 Live/dead cell viability assay

Cell viability was evaluated using the Calcein-AM/PI staining kit (Dojindo, C542). Samples were rinsed three times with PBS, immersed in a working solution containing 1 μM Calcein-AM and 2 μM Propidium Iodide, and incubated in the dark for 15 min. After washing with PBS (thrice), fluorescent images were obtained using a confocal microscope, and viability was quantified with ImageJ.

### 2.7 Immunofluorescence staining

Samples were fixed with 4% paraformaldehyde at room temperature for 30 min, rinsed three times with PBST (0.1% Tween 20), and permeabilized with 0.3% Triton X-100 for 15 min. After supplementary PBST washes, samples were blocked with blocking buffer (P0102, Beyotime) for 2 h at room temperature. Primary antibodies anti-CD31 antibody (Abcam, ab9498) and anti-E-cadherin antibody (Cell Signaling Technology, 3195s) were diluted in antibody dilution buffer and incubated overnight at 4 °C, followed by PBST washes and secondary antibody Donkey Anti-Rabbit IgG H&L (Alexa Fluor® 488) (Abcam, ab150073) and Goat Anti-Mouse IgG H&L (Alexa Fluor® 568) (Abcam, ab175473) incubation for 2 h at room temperature. After final washes, samples were imaged under a confocal microscope.

For quantitative analysis, the fluorescence intensity of the target protein was normalized to the fluorescence intensity of DAPI.

### 2.8 Cell seeding and culture in the chip

Fibrin hydrogels were prepared by mixing 8 mg/mL fibrinogen (Solarbio, F8050) with an equal volume of 4 U/mL thrombin (Solarbio, T8021) solution on ice. HUVECs (1.4 × 10^7^ cells/mL) and 3T3 fibroblasts (2 × 10^6^ cells/mL) were suspended in the thrombin solution and gently mixed with the fibrinogen solution. The mixture was promptly injected into culture chambers via perfusion inlets.

Chips were placed in a humidified incubator at 37 °C and 5% CO_2_ for 30 min. After fibrin polymerization, 100 μL of culture medium was added through medium inlets and replaced daily. For intestinal-chip construction, Caco-2 spheroids were additionally introduced at 4 × 10^4^ cells/ml.

### 2.9 Irradiation

Ionizing radiation was administered using a biological X-ray irradiator (RS2000, Rad Source Technologies, USA). The vascularized intestinal chips were exposed to a single dose of 8 Gy. During irradiation, chips were maintained under standard culture conditions and returned to the incubator immediately after exposure. Sham-irradiated controls underwent identical handling procedures without radiation exposure.

### 2.10 Flow cytometry

For analysis of co-cultured intestinal spheroids, samples were detached, centrifuged (300–400 g, 3 min), and dissociated into single cells using trypsin at 37 °C for 3 min. Suspensions were filtered through 40 μm cell strainers (Falcon, 352340) and stained with anti-E-cadherin antibody (Abcam, ab40772). Cell sorting and collection were performed using a MoFlo Astrios EQ flow cytometer (Beckman Coulter).

### 2.11 Quantitative RT-PCR

RNA from sorted epithelial cells was extracted using RNeasy Mini Kit (Qiagen). cDNA was synthesized with ReverTraAce (Toyobo). qRT-PCR was performed using TransStart Green qPCR SuperMix (TransGen Biotech) on a LightCycler 480 system (Roche). *GAPDH* was used as a reference, and relative gene expression was calculated with the ΔCt method from triplicate experiments.

### 2.12 Statistical analysis

Statistical analysis was carried out using GraphPad Prism (version 9.3.0, GraphPad Software, USA). Significant differences among groups were evaluated using Student’s t-test or one-way ANOVA. Data are expressed as mean ± standard deviation (SD), and p < 0.05 was considered statistically significant.

## 3 Results

### 3.1 Construction of cell spheroids using low-adhesion microwell plates

**FIGURE 2:**
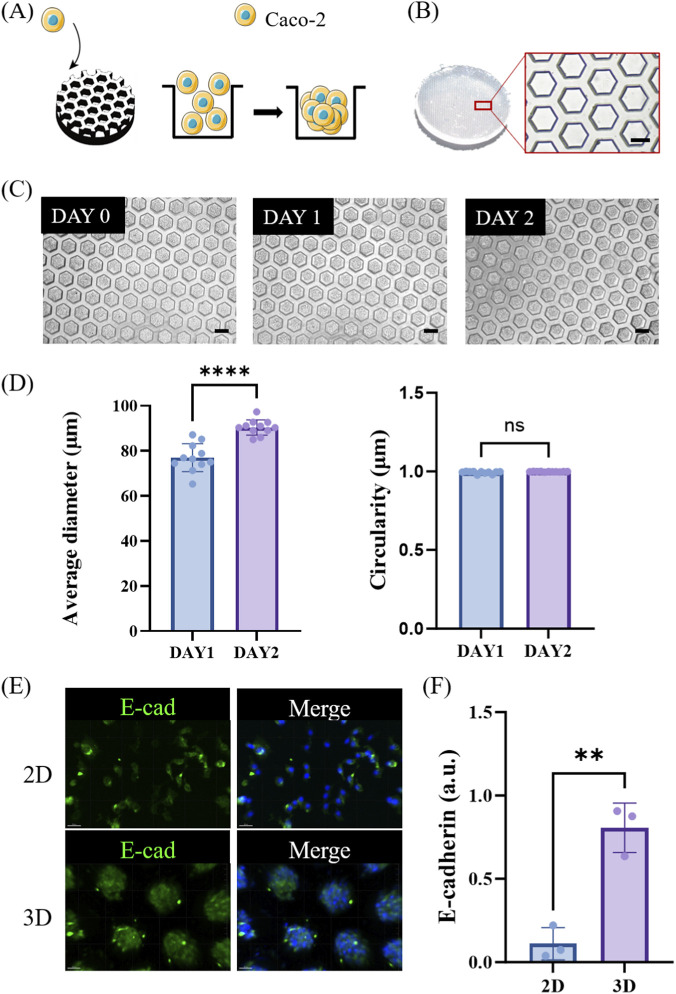
Low-adhesion microwell facilitate the formation of uniform cell spheroids and upregulate E-cadherin expression. **(A)** Low-adhesion microwell array for the formation of uniform multicellular spheroids. The hexagonal microstructure ensures consistent aggregate size and facilitates controlled cell assembly. **(B)** Photograph of the hydrophobic microwell array chip. The inset shows a magnified view of the hexagonal well pattern, demonstrating the ordered microstructure that facilitates spheroid formation. **(C)** Light microscopy images of Caco-2 cell clusters cultured in low-adhesion microwell plate on day 0, day 1, and day 2. **(D)** Mean diameter and circularity of Caco-2 cell clusters on days 1 and day 2 (n = 11, ****p < 0.0001). **(E)** Immunofluorescence staining of E-cadherin in 2D and 3D cultures, displaying uniform spheroid formation and stronger E-cadherin signals in low-adhesion microwell. **(F)** Quantification of E-cadherin fluorescence intensity (n = 3, **p < 0.01). Data represent mean ± SD **(D,F)**. The data were analyzed by unpaired t-test with Welch-correction **(D,F)**. Scale bar = 100 μm.

To establish a scaffold-free three-dimensional culture model, cells were seeded into low-adhesion microwell plates to enable spontaneous cell aggregation (Figure 2A). This platform was fabricated using soft lithography to generate arrays of concave microwells at the micrometer scale. Specifically, we first patterned a silicon wafer with a hexagonal-pillar array by photolithography, and the wafer morphology and dimensional characteristics are shown in Supplementary Figure S1. Using this master mold, PDMS was cast to replicate the geometry and form the hexagonal microwell array (Figure 2B). The geometrical confinement provided by the microwell architecture restricts cell spreading and migration, while the low-adhesive surface prevents cell attachment to the substrate. Figure 2C shows light microscopy images of Caco-2 cell clusters cultured in low-adhesion microwell on day 0, day 1 and day 2. As shown, Caco-2 cells formed uniform clusters within 24 h in the low-adhesion microwell plate and still exhibited stable cluster morphology at 48 h. Statistical analysis of the diameter and circularity of the Caco-2 cell clusters on days 1 and 2 (Figure 2D) revealed that the clusters on day 1 and day 2 were uniformly circular and nearly perfect circles. Furthermore, the diameter of the cell clusters on day 2 increased significantly, indicating robust proliferation of the Caco-2 cell clusters. These results demonstrate that using our platform for cell cluster culture enables the rapid and efficient formation of viable, uniform spheres with good reproducibility. To compare cellular morphology and adhesion characteristics under two-dimensional (2D) and 3D culture conditions, immunofluorescence staining was performed (Figure 2E). In conventional 2D culture, cells exhibited a flattened and dispersed morphology, with relatively weak E-cadherin expression that was localized only to limited regions of the cell membrane. In the three-dimensional culture system, E-cadherin exhibited a continuous circumferential distribution at cell–cell contact sites, indicative of strengthened intercellular adhesion. Quantitative analysis confirmed that E-cadherin fluorescence intensity was significantly higher in 3D-cultured cells compared with the 2D group (p < 0.01) (Figure 2F).

Collectively, these findings demonstrate that the microwell-based 3D microenvironment promotes spontaneous cell self-organization into uniform and stable spheroids, while enhancing cell–cell adhesion and supporting the establishment of epithelial barrier-related characteristics.

### 3.2 Construction of multicellular assembloids incorporating stromal components

**FIGURE 3:**
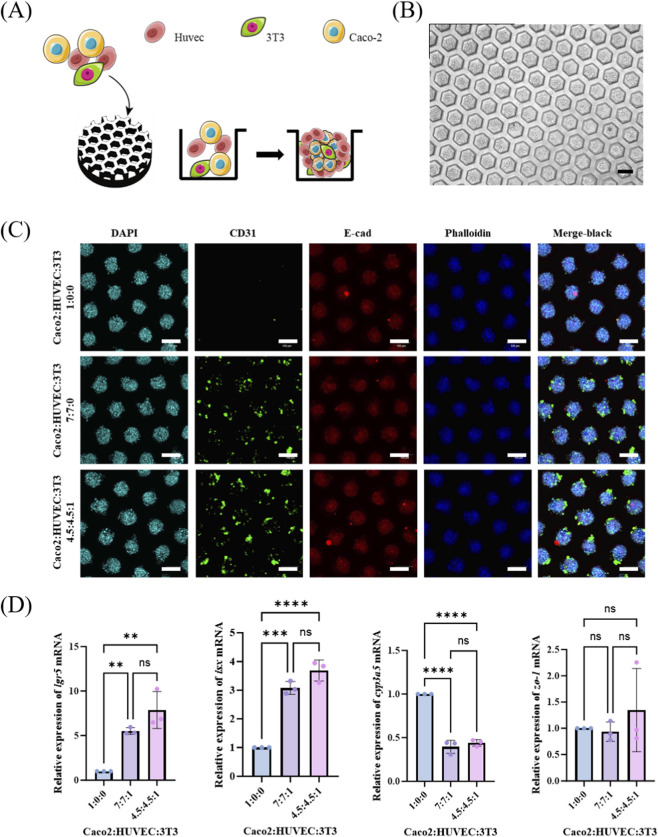
Multicellular assembloid formation within hexagonal microwell arrays. **(A)** Schematic illustration of the multicellular cultivation process. Caco-2 (intestinal epithelial), HUVEC (endothelial), and NIH-3T3 (fibroblast) cells were mixed at different ratios and seeded into hydrophilic hexagonal microwell arrays, where spatial confinement promoted spontaneous 3D aggregation and spheroid formation. **(B)** Light microscopic image showing co-cultured cells (Caco-2:HUVEC:3T3 = 4.5:4.5:1) distributed within microwells on day 2. **(C)** Immunofluorescence characterization of spheroids prepared with varied cell ratios (Caco-2:HUVEC:3T3 = 1:0:0, 7:7:1, and 4.5:4.5:1), labeled for CD31 (green, endothelial marker), E-cadherin (red, epithelial junctions), Phalloidin (blue, actin filaments), and DAPI (cyan, nuclei). **(D)** Quantitative analysis of relative mRNA expression levels of representative functional genes (*Lgr5*, *Isx*, *Cyp3a5*, and *Tff3*) in assembloids formed under different co-culture conditions, demonstrating enhanced epithelial differentiation and barrier-related gene expression under appropriate cell ratio (Caco-2:HUVEC:3T3 = 4.5:4.5:1) (n = 3, **p < 0.01, ***p < 0.001, ****p < 0.0001). Data are presented as mean ± SD **(D)**. The data were analyzed by One-way ANOVA with Tukey’s multiple comparison test **(D)**. Scale bar = 100 μm.

Building upon the established low-adhesion microwell system, we further developed a multicellular assembly strategy enabling the co-culture of epithelial cells, endothelial cells, and fibroblasts (Figure 3A). Caco-2, HUVEC, and 3T3 cells were mixed at defined ratios and seeded into low-adhesion microwell arrays. Within the confined microenvironment, cells spontaneously self-organized into multicellular spheroids, recapitulating key aspects of the coordinated epithelial–stromal–vascular architecture observed *in vivo* (Figure 3B).

To obtain structurally stable and organoid-like spheroids, we evaluated the effects of different cell ratios on tissue assembly. The ratios of Caco-2: HUVEC: 3T3 were adjusted to 1:0:0, 7:7:1, and 4.5:4.5:1 (Figure 3C). Immunofluorescence analysis showed that under these three conditions, cells formed compact assembloids. Furthermore, upon incorporation of HUVECs and 3T3 fibroblasts, the 3 cell types were uniformly distributed throughout the spheroids, indicating successful multicellular integration.

Gene expression analysis further demonstrated that, relative to Caco-2 monoculture spheroids, multicellular assembloids exhibited significant upregulation of epithelial stem cell marker *Lgr5*, intestine-specific transcription factor *Isx*, differentiation-related drug-metabolizing enzyme *Cyp3a5* (Figure 3D). Among the tested conditions, the 4.5:4.5:1 ratio yielded the highest expression levels of some functional markers, suggesting enhanced epithelial maturation and functional relevance.

Collectively, these results indicate that the engineered 3D multicellular co-culture system not only structurally recapitulates key features of intestinal tissue microarchitecture but also functionally enhances epithelial differentiation and physiological relevance through optimized cellular composition. This biomimetic platform provides a robust foundation for subsequent tissue modeling and toxicological applications.

### 3.3 Optimization of oxygen mass transfer to generate functionally enhanced assembloids

**FIGURE 4:**
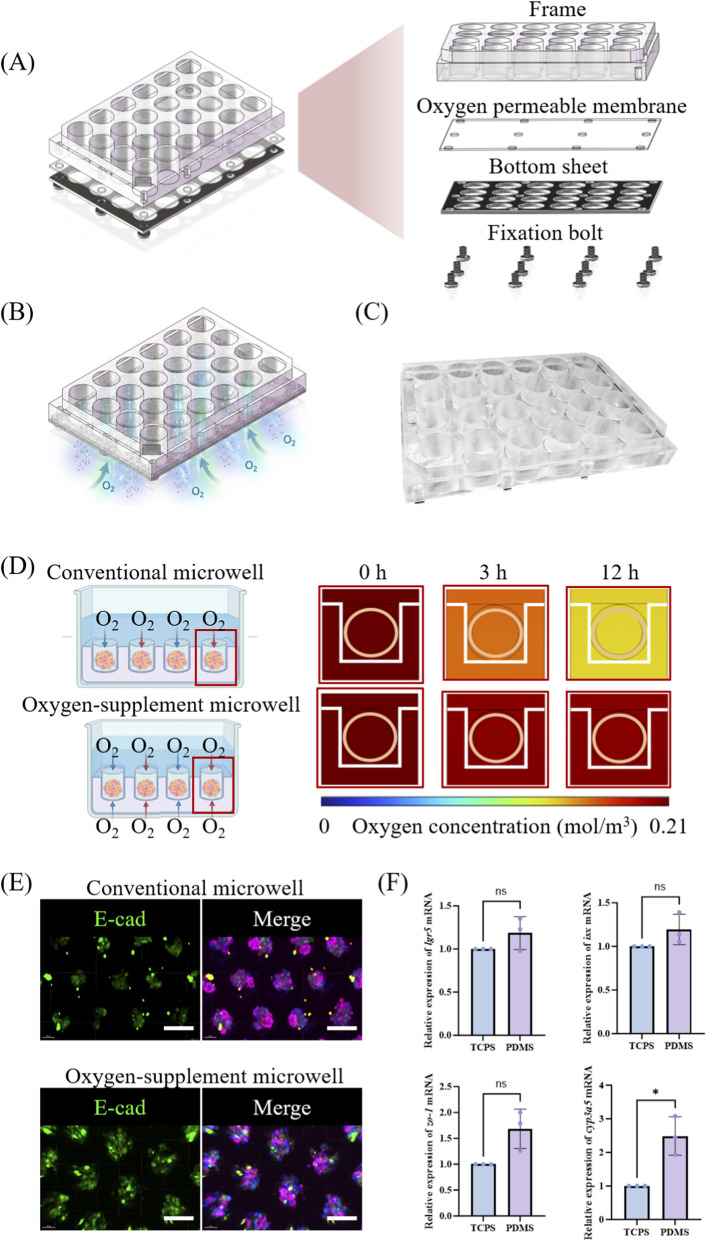
Oxygen-permeable culture plate and its effect on oxygen distribution and epithelial assembly. **(A)** Schematic illustration of the 24-well plate with a bottom oxygen-permeable membrane. The plate is assembled from a frame, oxygen-permeable PDMS membrane, bottom sheet, and fixation bolts to ensure oxygen delivery. **(B)** Oxygen-permeable multiwell plate used to enhance oxygen transfer and promote structural and functional maturation of intestinal spheroids. Arrows indicate oxygen diffusion through the PDMS membrane. **(C)** Photograph of the oxygen-permeable culture plate. **(D)** Numerical simulation of oxygen diffusion dynamics in conventional and oxygen supplemented microwells. Color maps show the predicted oxygen concentration surrounding cell aggregates at 0, 3, and 12 h, indicating enhanced oxygen concentration in the oxygen-permeable culture plate. **(E)** Fluorescence microscopy images of assembloids cultured under conventional and oxygen-supplemented conditions, immunostained for E-cadherin (green). Nuclei are stained with DAPI (blue), endothelial cells are labeled with CD31 (red), and F-actin is shown in pink. **(F)** Quantitative analysis of relative mRNA expression levels of representative functional genes (*Lgr5*, *Isx*, *ZO-1*, and *Cyp3a5*), demonstrating enhanced epithelial differentiation and barrier-related gene expression under oxygen-permeable conditions (n = 3, *p < 0.05). Data represent mean ± SD **(F)**. The data were analyzed by unpaired t-test with Welch-correction **(F)**. Scale bar = 100 μm.

To further improve oxygen supply during 3D spheroid formation, we developed a custom-designed 24-well plate with a polydimethylsiloxane (PDMS) bottom layer, matching the dimensions of standard commercial plates (Figure 4A; Supplementary Figure S2). Owing to the high oxygen permeability of PDMS, this design enables oxygen delivery not only through conventional diffusion from the culture medium but also directly from the bottom surface of the plate (Figures 4B,C). This dual oxygen supply pathway enhances overall oxygen mass transfer within the 3D culture system.

To evaluate the oxygen diffusion efficiency of the oxygen-permeable culture system, we compared it with the conventional tissue culture-treated polystyrene (TCPS) plate, which is non-permeable microwell plate. As shown in Figure 4D, in the conventional culture plate, oxygen can only diffuse downward from the medium above the cells, resulting in limited oxygen supply to the cell microenvironment. In contrast, the oxygen-supplement microwave design allows oxygen to permeate through the bottom membrane, facilitating bidirectional oxygen diffusion from both the upper medium and lower substrate toward the cells. Simulation analysis based on Fick’s first law of gas diffusion and Michaelis–Menten kinetics further indicated that, with increasing culture time, the oxygen concentration around assembloids in the oxygen-permeable plate remained significantly higher than that in the conventional plate (Figure 4D). These results demonstrate that the oxygen-permeable design effectively improves oxygen availability and establishes a more physiologically relevant culture environment.

To evaluate the biological impact of enhanced oxygenation, we compared spheroids cultured in the PDMS-bottom oxygen-permeable system with those formed in conventional oxygen-impermeable commercial plates containing low-adhesion inserts, as described above. Immunofluorescence analysis revealed that spheroids generated under oxygen-permeable conditions exhibited markedly stronger E-cadherin staining, indicating enhanced cell–cell adhesion and a more compact spheroid architecture (Figure 4E).

Consistently, quantitative PCR analysis demonstrated that the expression of genes associated with epithelial differentiation and function, including *Isx*, *Cyp3a5*, and *Tff3*, was upregulated in spheroids cultured under oxygen-permeable conditions compared to the non-permeable control, especially the expression of *Cyp3a5* (p < 0.05) (Figure 4F). These findings suggest that improved oxygen availability promotes epithelial maturation and supports the maintenance of physiological functionality in the 3D assembloids.

Taken together, the PDMS-based oxygen-permeable culture system effectively enhances oxygen mass transfer within the 3D model, leading to structurally and functionally improved multicellular spheroids. This optimized oxygenation strategy addresses a critical challenge in advanced *in vitro* systems and provides a more physiologically relevant platform for toxicological assessment and drug evaluation, aligning with current efforts to improve oxygen delivery in modern cell culture models.

### 3.4 Construction of a vascularized intestinal model

**FIGURE 5:**
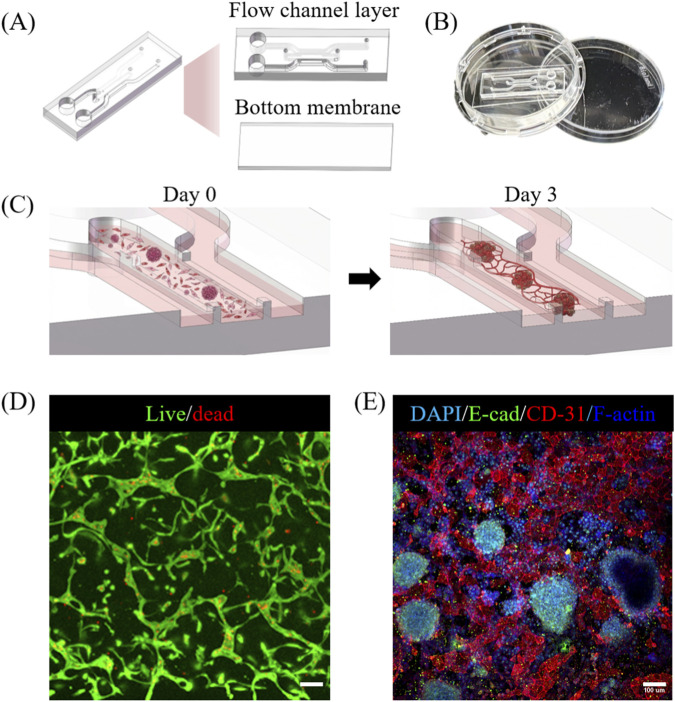
Design and characterization of the vascularized intestinal organ-on-a-chip. **(A)** Structural schematic of the microfluidic chip showing the layout of the cell and medium channels. **(B)** Photograph of the organ-on-a-chip. **(C)** Illustration of the dynamic formation of the vascularized intestinal model inside the chip. Initially, dispersed endothelial cells and intestinal assembloids were introduced into the microchannel; after a 3-day co-culture period, the endothelial cells self-organized into interconnected vascular networks that established physical contact with nearby intestinal spheroids. **(D)** Live/dead staining image displaying the formation of a viable and interconnected vascular network inside the chip. **(E)** Immunofluorescence staining showing the expression of E-cadherin (green) and CD31 (red), indicating the coexistence of intestinal spheroids and vascular structures within the chip. Scale bar = 100 μm.

To further enhance nutrient and gas exchange efficiency in the 3D tissue model, we developed a vascularized microfluidic chip with controllable microchannel architecture. The chip consists of upper and lower layers separated by a half-height barrier structure that defines a central hydrogel channel (Figures 5A,B; Supplementary Figure S3). Endothelial cells seeded within the hydrogel self-assemble into capillary-like microvascular networks, thereby recapitulating key aspects of *in vivo* vascular organization (Figure 5C).

Self-assembly of vascular endothelial cells within hydrogel matrices is a commonly used approach for constructing *in vitro* vascularized tissue models (Chen et al., 2017; Liu et al., 2025). Briefly, endothelial cells were suspended in a hydrogel matrix and introduced into the designated gel channel. At day 3 of culture, the morphology and viability of endothelial cells within the chip were evaluated. Live/dead staining revealed that most cells were viable, as indicated by prominent green fluorescence, whereas only weak red fluorescence corresponding to dead cells was detected (Figure 5D).

Subsequently, the pre-established 3D intestinal epithelial spheroids were integrated into the vascularized chip to construct a vascularized intestinal model. Immunofluorescence analysis revealed that E-cadherin-positive intestinal epithelial spheroids (green) were spatially closely associated with CD31-labeled vascular networks (red) (Figure 5E). In certain regions, direct contact and partial envelopment between the epithelial spheroids and vascular structures were observed, indicating the successful establishment of an *in vitro* intestinal model with integrated vascular networks.

Together, these findings demonstrate that the engineered microfluidic platform not only supports vascular network formation but also enables co-culture of vascular and intestinal epithelial components. This vascularized intestinal chip establishes a more favorable oxygen transport environment for assembloids, and provides a physiologically relevant platform for modeling the intestinal–vascular interface *in vitro* and offers enhanced potential for toxicological and pharmacological investigations.

### 3.5 Evaluation of radioprotective agents using the vascularized intestinal chip

To assess the applicability of the vascularized intestinal chip for modeling radiation-induced injury and pharmacological intervention, the established platform was exposed to ionizing radiation. This was followed by evaluation of the candidate radioprotective agent dimethyloxaloylglycine (DMOG), which has been shown to mitigate intestinal radiation injury when administered prior to irradiation. DMOG was administered at a final concentration of 1 mM, 12 h prior to irradiation.

**FIGURE 6:**
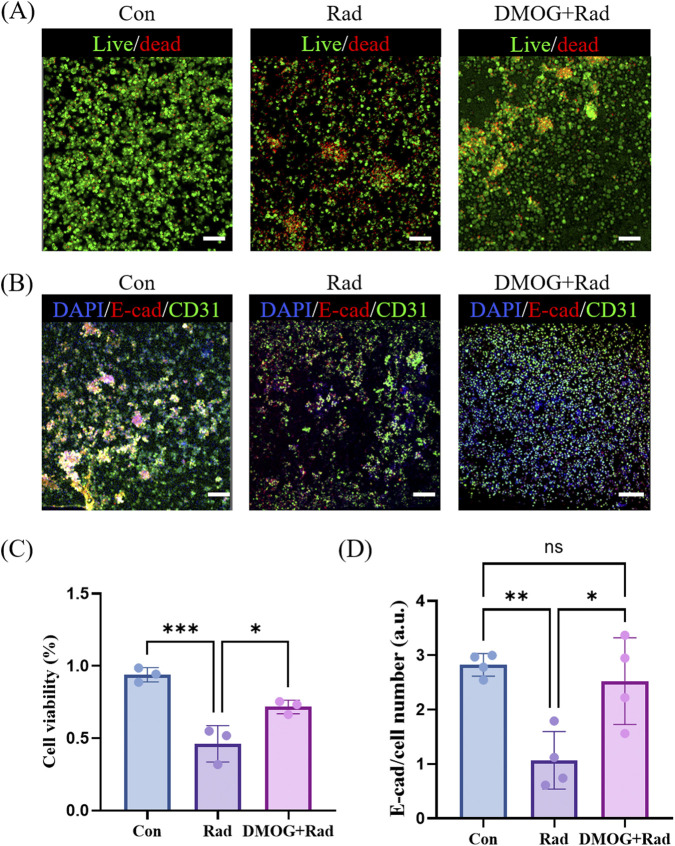
Assessment of radiation-induced injury and drug evaluation on the vascularized intestinal chip. **(A)** Live/dead staining of the control, irradiated, and drug-treated + irradiated groups, showing changes in cell viability after radiation exposure and pharmacological intervention. **(B)** Immunofluorescence staining of the control, irradiated, and drug-treated + irradiated groups, with DAPI (blue), E-cadherin (red), and CD31 (green) labeling nuclei, intestinal epithelial cells, and endothelial cells, respectively. **(C)** Quantitative analysis of live/dead staining indicating a significant decrease in cell viability after irradiation, and partial recovery in the drug-treated group (n = 3, *p < 0.05, ***p < 0.001). **(D)** Quantitative analysis of E-cadherin fluorescence intensity showing epithelial damage after irradiation, with drug treatment mitigating the radiation-induced loss of epithelial function (n = 4, *p < 0.05, **p < 0.01). Data are presented as mean ± SD **(C,D)**. The data were analyzed by One-way ANOVA with Tukey’s multiple comparison test **(C,D)**. Scale bar = 100 μm.

Live/dead staining demonstrated a marked increase in red fluorescence after irradiation, indicating substantial cell death and structural disruption in both the intestinal epithelial spheroids and the vascular network (Figure 6A). Quantitative analysis of cell viability (Figure 6C) confirmed a significant reduction in survival in the irradiated group compared to the non-irradiated control. Pre-treatment with DMOG partially restored cell viability, indicating a protective effect against radiation-induced damage.

Immunofluorescence analysis further revealed that irradiation led to a substantial decrease in the expression of the epithelial marker E-cadherin, accompanied by disruption of intercellular junctions (Figure 6B). In the DMOG-treated group, E-cadherin expression showed partial recovery, and the continuity of epithelial spheroids was better preserved. Quantitative analysis of E-cadherin fluorescence intensity (Figure 6D) was consistent with these observations: irradiation significantly reduced E-cadherin levels, whereas DMOG treatment partially restored its expression, suggesting mitigation of radiation-induced epithelial damage and partial preservation of tissue structural integrity.

Collectively, the vascularized intestinal chip successfully recapitulated key features of radiation-induced intestinal injury and enabled evaluation of radioprotective intervention. DMOG demonstrated a measurable protective effect in maintaining cell viability and epithelial integrity, further supporting the utility of this oxygen-optimized, vascularized *in vitro* platform for toxicological and therapeutic assessment.

## 4 Discussion

The intestinal epithelium plays a pivotal role in nutrient absorption, immune defense, and xenobiotic metabolism, making it an important subject in toxicology and radiobiology model development. However, the high radiosensitivity and strong metabolic demands of intestinal tissue pose significant challenges to establishing stable and physiologically relevant *in vitro* models. Recent advances in three-dimensional culture and microphysiological platforms have sought to reproduce the complex cellular composition and oxygen transport characteristics of intestinal tissue, yet existing models often suffer from simple cellular composition, insufficient oxygenation, and lack of vascular components (Jalili-Firoozinezhad et al., 2019; Shen et al., 2020; Wang et al., 2025). The present study addressed these limitations through a multilayered strategy incorporating controllable multicellular assembly, enhanced oxygen delivery, and vascular integration to construct a more biomimetic intestinal tissue model.

At the cellular level, the incorporation of mesenchymal and endothelial cells alongside epithelial cells was critical not only for improving physiological performance but also for modeling radiation-induced intestinal injury. In radiation enteropathy, endothelial apoptosis and microvascular dysfunction represent early and decisive events that amplify epithelial damage. Such mechanisms cannot be recapitulated in epithelial monocultures (Paris, Fuks, Kang, Capodieci, Juan, Ehleiter, Haimovitz-Friedman, Cordon-Cardo and Kolesnick, 2001; Wang et al., 2007). Fibroblasts, as key mesenchymal components, not only provide mechanical support but also modulate epithelial proliferation, differentiation, and polarization through secretion of extracellular matrix proteins (e.g., fibronectin) and growth factors such as BMP, WNT, and TGF-β (Chen et al., 2024; McCarthy et al., 2020; Yetkin-Arik et al., 2024). Based on these well-established stromal functions, we selected 3T3 fibroblasts as a representative and well-characterized mesenchymal cell line to establish a stable and reproducible supportive compartment within the engineered microenvironment (Montesano et al., 1993, Nishiguchi et al., 2014; Wang et al., 2017). Although 3T3 cells are of murine origin, they are widely used for their robust extracellular matrix–producing capacity and consistent growth characteristics. In the present study, they were incorporated to provide general stromal support rather than to investigate species-specific signaling mechanisms. Endothelial cells supported oxygen and nutrient exchange and secreted angiocrine cues that facilitated epithelial repair (Potente and Mäkinen, 2017; Rafii et al., 2016). Within the hexagonal microwell platform, controlled-ratio co-culture of Caco-2, HUVEC, and 3T3 cells produced compact spheroidal structures with uniform morphology and upregulated genes related to stemness and metabolism (*Lgr5*, *Isx*, *Cyp3a5*). These findings highlight that proper mesenchymal–epithelial interactions are essential for epithelial maturation and function, emphasizing reconstruction of native cell microenvironmental cues.

Oxygen delivery efficiency represents another critical determinant of cell functionality in 3D systems and is particularly relevant in radiation studies. Diffusion-limited hypoxia in conventional spheroids may independently alter ROS homeostasis and epithelial barrier integrity prior to irradiation, thereby confounding interpretation of radiation-induced oxidative damage (He et al., 2022). Dense cell aggregates restrict diffusion, leading to localized hypoxia and impaired barrier function (He et al., 2022; McMurtrey, 2016; Mosaad et al., 2018; Potente and Mäkinen, 2017). By introducing an oxygen-permeable PDMS base, the culture platform provided bidirectional diffusion from both upper and lower interfaces, markedly improving oxygen distribution and enhancing adhesion and differentiation markers. Simulation and experimental data consistently indicated that adequate oxygen availability promotes continuous expression of adhesion molecules such as E-cadherin and upregulates intestine-specific functional genes, consistent with prior studies on oxygen-regulated epithelial functional performance (Nolfi-Donegan et al., 2020; Pral et al., 2021). This improved micro-oxygen environment resolves the common metabolic and hypoxic constraints encountered in 3D cell spheroid and organoid culture.

Further vascularization enhanced nutrient exchange and gas diffusion. In the microfluidic chip, endothelial cells spontaneously assembled into microvessel networks within a fibrin hydrogel and coexisted with epithelial aggregates, thereby more closely recapitulating the intestinal–vascular interface. Immunofluorescence revealed that CD31-positive endothelial networks were closely aligned and often intertwined with E-cadherin-positive epithelial structures, confirming successful vascularized model construction.

Under radiation exposure, the chip exhibited marked radiosensitivity, including increased cell death, reduced E-cadherin expression, and structural disruption—findings consistent with clinical radiation enteropathy (Shadad et al., 2013; Singh et al., 2015). Pretreatment with the hypoxia-inducible factor stabilizer DMOG significantly mitigated epithelial injury and partially restored structural integrity, demonstrating the suitability of this model for evaluating radiation damage and pharmacological protection.

## 5 Limitations and future directions

Although this study developed a multicellular intestinal assembloid integrating epithelial, endothelial, and mesenchymal components with improved oxygenation, it still represents a simplified microenvironment. Key physiological cues such as neuro cells, immune regulation, and microbiota interactions were not included, though these play critical roles in epithelial homeostasis and injury response (Jacobson et al., 2021; Múnera et al., 2023; Xie et al., 2026). Incorporating immune cells or microbial metabolites in future designs may further enhance physiological relevance. Another limitation lies in the use of immortalized cell lines, which do not fully represent primary human tissue heterogeneity. Employing iPSC-derived or patient-specific intestinal cells could improve translational fidelity and enable personalized toxicity or radiation-response studies (Clevers, 2026). In addition, the present model employed murine-derived 3T3 fibroblasts in combination with human Caco-2 epithelial cells, resulting in an interspecies co-culture system. While 3T3 fibroblasts are widely used due to their stable growth characteristics and well-established roles in growth factor secretion and extracellular matrix production, species-specific differences may influence certain ligand–receptor interactions. Future studies will transition to human primary intestinal fibroblasts or a human intestinal fibroblast cell line (e.g., CCD-18Co) to further enhance physiological relevance. The current setup also lacks mechanical stimulation—cyclic strain and luminal flow—that influences barrier maturation and vascular integrity (Kim et al., 2012). Integrating dynamic perfusion or stretchable membranes would better emulate peristaltic conditions. Finally, the current work focused on testing a compound previously reported to exert radioprotective effects. Building upon this proof-of-concept, future studies could parallelize multiple intestinal chips to achieve high-throughput screening of additional candidate drugs with potential therapeutic or protective efficacy against irradiation. Overall, enhancing cellular diversity, physiological realism, and experimental throughput will further expand the application of intestinal assembloid-on-chip models for high-throughput drug screening, toxicity testing, and radiation-injury research.

## Data Availability

The original contributions presented in the study are included in the article/Supplementary Material, further inquiries can be directed to the corresponding author.
