# Supplementary material for: Oxygen-enhanced assembloid-based vascularized intestinal-on-a-chip for radioprotective drug evaluation
Source: Front Toxicol. 2026 Jul 16;8:1863392. doi: 10.3389/ftox.2026.1863392 (PMC13421179; doi:10.3389/ftox.2026.1863392)
Supplement: Supplementary file 1 [file DataSheet1.docx]

Supplementary Material

# Supplementary Figures


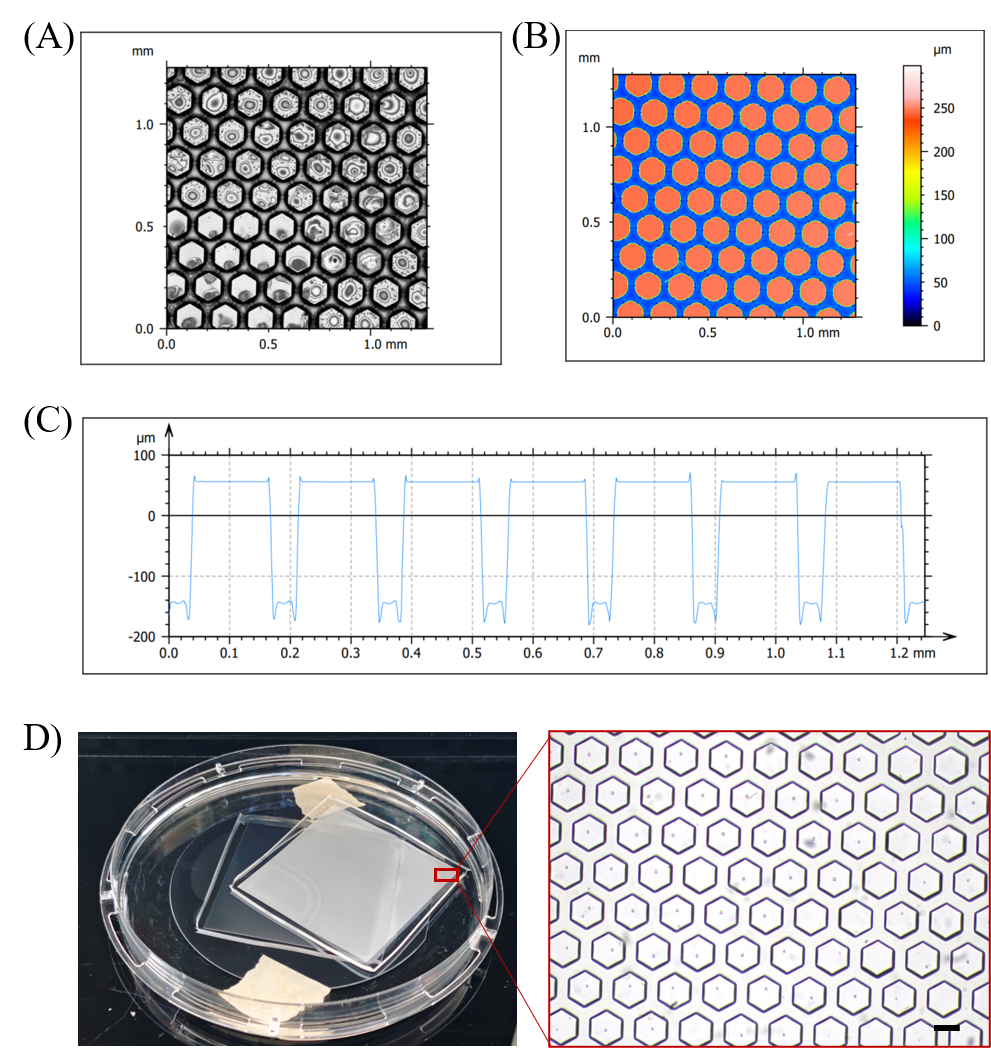


**Supplementary Figure 1.** Morphological characterization and fabrication of the hexagonal microwell array substrate. (A) Confocal microscopic image of the patterned silicon master showing regularly arranged hexagonal micropits. (B) Surface topography map of the silicon template obtained by 3D profilometry, with color scale representing height variation across the patterned area. (C) Height–position profile of the silicon surface extracted from the topography data, confirming uniform feature depth and periodicity along the measured line. (D) Soft‑lithography fabrication of the PDMS film carrying the hexagonal microwell array. The photograph shows the replicated PDMS layer placed in a Petri dish, and the magnified view highlights the precise reproduction of the hexagonal micropattern.


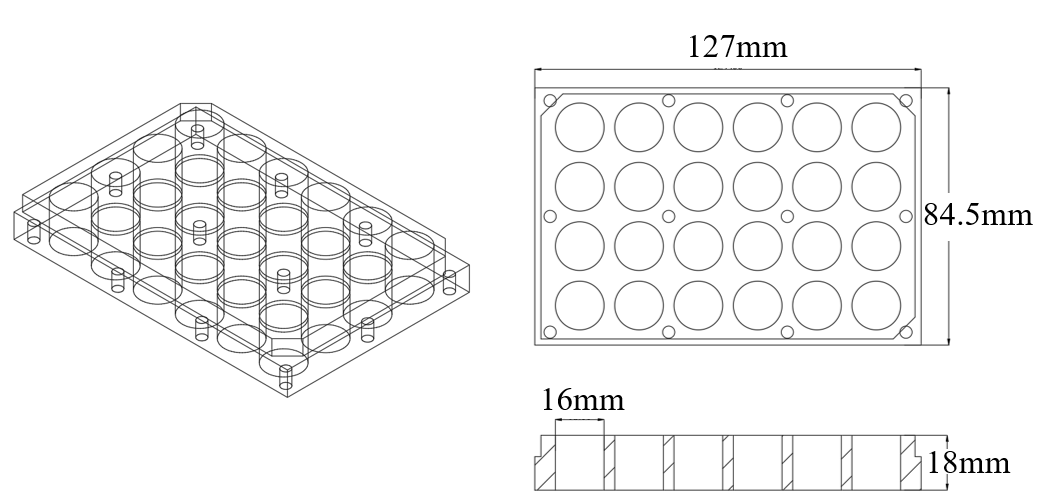


**Supplementary Figure 2.** Schematic design and dimensions of the oxygen-permeable 24-well platform.


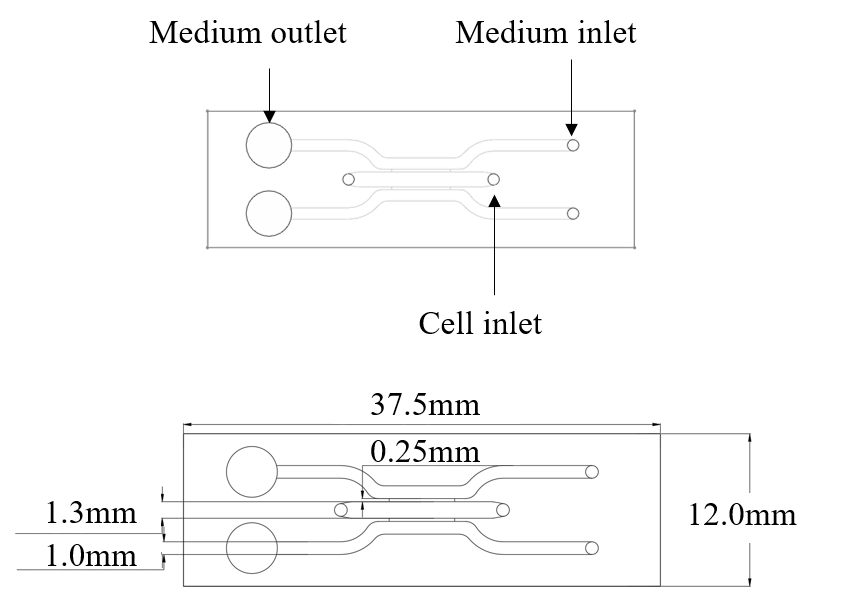


**Supplementary Figure 3.** Schematic design and dimensions of the microfluidic chip.
